# Family Dialogues on Sexuality: A Contingential Analysis of Gender, Care, and Mother–Adolescent Children Communication

**DOI:** 10.3390/healthcare14020251

**Published:** 2026-01-20

**Authors:** Angel de Jesús Angulo Moreno, Abner Daniel Ramírez Arzate, María Dolores Aragón Robles Linares

**Affiliations:** 1 Research Monitoring and Dissemination Department, Central Office, Servicios de Salud del Instituto Mexicano del Seguro Social para el Bienestar (IMSS-BIENESTAR), Av. Insurgentes Sur 1940, 9th Floor, Col. Florida, Álvaro Obregón, Mexico City 01030, Mexico; 2 Department of Social Sciences, Universidad de Sonora, Nogales 83000, Mexico; dolores.aragon@unison.mx

**Keywords:** comprehensive sexuality education, interbehavioral psychology, adolescence

## Abstract

From an interbehavioral and contingential perspective, family dialogues about sexuality are understood as patterns of verbal interaction regulated by social, gender, and caregiving contingencies rather than as individual attitudes or intentions. **Background:** This study analyzes the functional conditions under which family dialogues about sexuality occur between mothers and their adolescent sons and daughters, considering caregiving roles and gender norms that regulate these interactions. The research aimed to identify the functional relations between communicative practices and the social contingencies that maintain or inhibit them. **Methods:** A qualitative approach grounded in interbehavioral psychology was employed, using semistructured interviews with 40 mothers of students from a public middle school in Puebla, Mexico. Data were analyzed through contingency analysis, distinguishing micro- and macrocontingential systems related to family sexual education. **Results:** Results show that, although patterns of avoidance and discourse displacement toward schools or peers persist, families exhibit increasing openness toward comprehensive sexuality education and recognize its preventive value against violence, adolescent pregnancy, and misinformation. Functional delegation and adolescent mediation of dialogue were identified, along with emerging inclusive macrocontingencies linked to the acceptance of diverse families and LGBTIQ+ themes. **Conclusions:** It is concluded that households function as self-regulated interbehavioral systems in which historical and gender contingencies restrict sexual dialogue, yet gradual functional changes toward respect, inclusion, and shared educational responsibility are observed.

## 1. Introduction

During the development of children and adolescents, diverse conditions of interaction and learning are established that orient sexuality and affective behavior. When opportunities for dialogue about sexuality are restricted in family and school environments, adolescents tend to turn to informal sources such as the Internet, where the contingencies of exposure often maintain imprecise or biased information, generating verbal repertoires that are ineffective for self-care [1,2].

Comprehensive sexuality education constitutes a system of interinstitutional educational practices aimed at generating social conditions for the guidance and regulation of sexuality. International evidence highlights that its effectiveness depends on the joint participation of educational agents, the community, and families [3]. However, in many contexts, family interactions regarding sexuality remain under the control of implicit norms [4], euphemisms, or verbal avoidance, reducing the transmission of functional repertoires necessary for the responsible exercise of sexuality [5].

The absence of functional educational and communicative practices increases children’s and adolescents’ exposure to risk contingencies associated with violence, exploitation, or misinformation. These phenomena are explained not by individual variables but by systems of social and normative control that define what can be said, permitted, or prohibited concerning sexuality [6]. Within this framework, Comprehensive Sexuality Education with a gender perspective and a progressive autonomy approach should be understood as a set of planned contingencies designed to reduce inequalities and reorganize the systems of social control that sustain discrimination [7].

In Mexico, the social macrocontingencies linked to gender inequality and institutional omission are reflected in indicators of fertility, adolescent pregnancy, and the prevalence of sexually transmitted infections [8,9,10]. Data show that sexual education patterns are mediated by educational level, family values, and religious frameworks, forming a complex system of normative control. Structural violence, expressed through femicides and the exclusion of sexual and gender diversity [8,11,12], confirms the need to analyze family practices as functional units within these social systems.

Even though institutional programs oriented toward inclusion and equity have been implemented [13], sexual education practices remain regulated by traditional values that limit family participation. Recent literature emphasizes the need to incorporate a contextual analytical approach to describe how dialogues about sexuality are established, maintained, or avoided within households [1,14,15].

Prior studies show that family interactions about sexuality are shaped by norms, avoidance patterns, and a lack of instructional repertoires. Systematic reviews indicate that communicative avoidance, parental discomfort, and reliance on external agents significantly modulate adolescents’ sexual learning [2,15]. Research in Mexico and Latin America documents that parents often prioritize biological risk prevention while limiting dialogue about identity, pleasure, or affective dimensions [16,17]. Likewise, studies with Indigenous and urban youth report that gender norms and moral frameworks strongly regulate what can be said at home, leading adolescents to depend on peers, school, or digital media for information [18]. These findings underscore the need to analyze family dialogue as a functional system influenced by social contingencies.

From this perspective, the verbal behavior of mothers, fathers, and caregivers constitutes a critical variable, as it functions as a mediator between social norms and school learning. Studying this behavior allows for the identification of regularities in the control systems that define the conditions for teaching and communicating about sexuality [19,20].

However, previous research in Mexico and Latin America has focused primarily on school or institutional settings, leaving the functional conditions of family interaction scarcely described. Consequently, there remains a gap in understanding how the systems of contingencies that maintain or inhibit family dialogue about sexuality are configured, particularly in contexts of vulnerability and gender normativity.

Under this logic, the present study aims to describe the functional regularities of mothers’ verbal behavior in family contexts related to the sexual education of their adolescent sons and daughters, identifying the normative control systems and caregiving roles that regulate these interactions. To this end, contingential analysis [21] is employed as the theoretical–methodological framework, allowing for the characterization of microcontinental systems (situated interactions) and macrocontingential systems (normative and value structures) that constitute the interbehavioral field of family sexuality education in the contemporary Latin American context [22]. Although the study initially aimed to include both mothers and fathers, the empirical absence of male participation reflects the gendered distribution of caregiving and communication roles in the community; therefore, the analysis focuses on mothers as the primary agents of family dialogue.

## 2. Materials and Methods

### 2.1. Context

The study was conducted in the residential complex Infonavit San Bartolo, located in Puebla, Mexico, considered a social interaction field characterized by structural vulnerability, unequal access to services, community violence, and limited water resources. This context operates as an antecedent macrocontingency that establishes the probabilities of occurrence for family communicative practices related to sexual topics. For example, when a mother expressed embarrassment, the interviewer’s prompts functioned as discriminative stimuli that either facilitated or inhibited elaboration. The interview was therefore treated as a dynamic field in which verbal behavior was shaped by immediate social contingencies. This context functions as an antecedent macrocontingency because its structural conditions, violence, limited services, and economic vulnerability shape the likelihood that certain communicative behaviors (e.g., avoidance, delegation, or openness) will occur during sexuality-related interactions within families.

The community of San Bartolo is characterized by strong moral norms influenced by Catholic traditions and local community leadership.

The community has 9770 residents distributed across 2510 households (density of 3301 inhabitants/km^2^), with an average age of 27 years and a mean schooling level of 10 years. Among its residents, approximately 3000 are under 14 years old, 4000 are between 15 and 29, and 490 are over 60 [23]. These conditions define a social system in which intergenerational verbal transmission is modulated by economic, institutional, and value contingencies.

### 2.2. Participants

A total of 40 adult women participated in the study, all of whom had a parental or caregiving relationship with second-grade students from a public technical middle school. The participation was voluntary, and only those who agreed to take part after receiving information about the study and confidentiality conditions were included.

Of the 40 participants, 92.5% were biological mothers, 6.25% were guardians, and 1.25% were aunts. Ages ranged from 21 to 55 years, with a predominance between 30 and 49 years. In four cases, participants verbally reported having experienced motherhood during adolescence. Although initial recruitment efforts included both mothers and fathers, participation from male caregivers was absent. This pattern reflects the gendered distribution of caregiving and communicative responsibilities in the community, where mothers assume the primary role in education and dialogue related to sexuality. Because of this empirical condition, the study focused on mothers as the main functional agents in the family interaction system.

The participants were forty mothers between 21 and 55 years of age (M = 38.4). Most were married (47.5%), followed by those who were single (22.5%), separated (20%), or reported another marital status (10%). Regarding education, 12% had completed primary school, 45% secondary school, 30% high school, and 13% had higher education. In terms of occupation, 55% identified as homemakers, 30% worked in informal employment, and 15% held formal jobs. Almost all participants reported a Catholic religious affiliation (97.5%), with a small proportion identifying as Christian (2.5%). All mothers self-identified as belonging to a low socioeconomic level. The number of children per participant ranged from one to four (M = 2.3). Their adolescent sons and daughters, the focus of the study, were between 12 and 14 years old.

### 2.3. Theoretical–Methodological Framework

The study was developed under a qualitative approach grounded in interbehavioral psychology, aimed at describing functional regularities in the systems of verbal interaction between mothers and adolescents in family contexts. The contingential analysis proposed by Rodríguez [24] was used as the theoretical–methodological system to identify functional relations among communicative practices, situational antecedents, and the social consequences that maintain them.

From this perspective, each interview was considered an interbehavioral field composed of verbal events between the researcher and the participant, where behavior was analyzed in terms of morphology, functionality, and value field (Table 1. Structure of the Interview Guide). Verbal behavior was recorded as a contextual event determined by antecedent contingencies (dialogue situations about sexuality), responses (verbal forms, avoidance, descriptions), and consequences (normative or familial reactions).

The analysis was structured at two levels: the microcontinental system, which includes situated verbal interactions and observable behaviors, and the macrocontingential system, encompassing the normative, cultural, and gender frameworks that function as dispositional variables regulating verbal behavior about sexuality.

During the initial phase of the study, when group situations were used, a different pattern of tendencies was observed. The presence of community leaders functioned as a dispositional factor in social behavior, modulating acceptance or non-acceptance of sexual education. The verbalizations of disapproval expressed by these leaders (“sexuality should only be taught at home”) generated collective adherence responses, reinforced and maintained by group approval. The social system thus operated as a social positive reinforcement contingency, in which agreement and conformity sustained behaviors of denial or censorship. Subsequently, when the study shifted to an individual interview format, the function of the stimuli changed: the same participants emitted verbal responses of acceptance toward sexual education. The reduction in discriminative stimuli diminished group control and allowed the emission of verbalizations more consistent with each participant’s personal history of interaction. María, for instance, stated: “I supported Juana in saying that sexuality should not be taught at school so as not to make her angry, because she is a leader and helps me with my daughter; but in fact, I do agree,” illustrating how the social context influences the form and frequency of verbal behavior in the field of sexuality. Before beginning the interview, participants were informed that ‘sexuality’ would be understood in a broad educational sense, including body development, emotions, gender, relationships, sexual health, rights, and preventive care, following the UNESCO comprehensive sexuality education framework. Comprehensive sexuality education, as defined by UNESCO [3], is a structured educational approach that integrates biological, emotional, social, and rights-based dimensions of sexuality. Its framework provides conceptual clarity for analyzing which components families adopt, avoid, or delegate to schools. In this study, comprehensive sexuality education functions as the evaluative reference to identify the alignment or discrepancy between maternal discourse and evidence-based sexuality education.

The interviews were conducted between January and June 2023 in open spaces adjacent to the middle school, ensuring privacy and the absence of contextual interference. Participants were informed about the purpose of the study, the conditions of participation, and the institutional procedures for a non-disclosure agreement.

Each interview was considered an interbehavioral field, in which the interviewer and the participant constituted interacting systems regulated by institutional and social norms. Verbalizations were audio-recorded, transcribed verbatim, and cross-checked with field notes.

The contingential analysis was conducted in three phases:

Morphological level: identification of the forms and sequences of verbal behavior.

Functional level: description of the relationships among antecedents, responses, and consequences.

Evaluative or macrocontingential level: detection of rules, norms, and value systems maintaining the observed behaviors.

An analytical pairing strategy was applied, in which two researchers independently coded the verbal units and reached consensus on emerging categories. Relative frequencies were represented as functional quantifications, aiming to visualize the recurrence of patterns without losing contextual interpretation.

### 2.4. Rigor Criteria and Ethical Considerations

The study was conducted in accordance with national ethical principles, understood as normative systems that regulate research behavior. Participation was anonymous and voluntary, and no physical or psychological risks were involved. Confidentiality was ensured through the aggregated presentation of data and the institutional safeguarding of all records.

The functional credibility of the analysis was maintained through peer review, correspondence between field notes and transcripts, and consensual validation of emergent categories. The entire procedure was systematically documented, ensuring traceability and coherence throughout all phases of the study.

## 3. Results

The analysis yielded eleven functional categories organized into two levels of contingential relations: microcontingential systems, which describe immediate interactional patterns between mothers and their adolescent children, and macrocontingential systems, which reflect the cultural, normative, and institutional conditions regulating those interactions. The categories identified were (1) maternal initiative to address sexuality, (2) adolescent mediation of dialogue, (3) avoidance and inhibitory patterns, (4) reference and substitution agents, (5) situations that facilitate dialogue, (6) value frameworks surrounding sexuality, (7) topics perceived as difficult to address, (8) priority themes for sexual education, (9) agreement with comprehensive sexuality education components, (10) age expectations for initiating comprehensive sexuality education, and (11) family-level contingential structures.

These categories are presented in the following subsections. Percentages reflect the relative frequency of coded verbal units associated with each functional category across the 40 interviews. Frequencies were calculated by identifying and counting discrete response units within each thematic code, allowing a descriptive estimation of the recurrence of specific communicative patterns. All participant names used in the results are pseudonyms to ensure confidentiality.

### 3.1. Functional Field of Family Interaction

Before presenting the specific categories emerging from the functional analysis, it is necessary to outline the general interactional context in which the participants’ verbal behavior occurs. The interviews revealed a family system shaped by culturally regulated gender roles, caregiving responsibilities, and social norms that influence how dialogue about sexuality is initiated, avoided, or delegated. This contextual characterization provides the foundational conditions under which the subsequent micro- and macrocontingential patterns are observed.

The analysis of the interviews made it possible to describe a system of interbehavioral relations in which mothers, as primary agents, act under conditions of social control that regulate their verbal behavior concerning sexuality.

The general interactional context is shaped by culturally regulated gender roles and social norms. The predominant family structure was the nuclear model (48.7%), followed by extended or reconstituted families (28.8%) and female single-parent households (22.5%). Notably, while one mother identified as lesbian living with her female partner, the majority of participants reported traditional gender roles and a heterosexual orientation. This configuration provides the foundational field where mothers, as primary agents, act under social control systems that regulate their verbal behavior concerning sexuality.

### 3.2. Initiative and Management of Sexuality Dialogue

To understand how sexual communication emerges within the family, it is necessary to examine the functional conditions under which mothers initiate or manage conversations about sexuality. This category captures the antecedents that evoke dialogue, the verbal responses mothers emit, and the consequences that maintain or inhibit these interactions. The findings reveal a pattern of irregular, situationally controlled exchanges shaped by norms of responsibility, embarrassment, and social evaluation, rather than by stable instructional practices. The main functional tendencies are presented below.

While 73% of mothers stated they had addressed sexuality on their own initiative—primarily motivated by the prevention of pregnancy or STIs—this does not reflect a consistent educational pattern. Instead, dialogue emerges reactively to external contingencies such as television programs or family gatherings. When these situations arise, a recurrent functional pattern of verbal avoidance (topic change or postponement) appears in 40% of cases.

This tension is evident in testimonies like Fernanda’s, who noted that “these are topics that are hard for me to discuss because there is a lack of trust and I feel embarrassed.” Such responses reveal a system of contradictory contingencies: the cultural “responsibility” to speak vs. the social punishment of “embarrassment.” Consequently, mothers often seek a transfer of instructional control to external agents. As Alica suggested, they prefer “someone who knows more,” illustrating a delegation of the educational role.

Despite these difficulties, affirmative verbalizations focused on protection predominated. For instance, Maria, Mara, and Melisa coincided in that dialogue is essential “to prevent diseases,” “provide information,” and ensure “the protection of the child.” These expressions configure a functional field where the primary objective is avoiding negative consequences—such as “unwanted pregnancies” or “misleading information”—rather than establishing stable instructional practices.

### 3.3. Initiative and Reactivity of Daughters and Sons

A key component of the family communication system is the extent to which adolescents initiate, inhibit, or redirect conversations about sexuality. Their verbal behavior functions as a discriminative stimulus that determines when dialogue occurs, how it unfolds, and whether it is sustained or avoided. This section examines the functional conditions under which daughters and sons mediate interactions with their mothers, highlighting patterns of communicative avoidance, situational openness, and delegation to external sources such as school or digital media. The findings reveal how adolescent reactivity operates as a central regulator of the family’s instructional possibilities.

The adolescent’s behavior functions as a central discriminative stimulus that regulates the family’s instructional possibilities. Most mothers (57%) reported that their children do not initiate questions, often due to socially conditioned avoidance responses. Tania and Isabel observed that while some seek to “know the consequences of irresponsibility,” others perceive the topic as “not yet relevant.”

Mothers described a recurrent pattern of inhibited exchange where “shame,” “insecurity,” and “lack of confidence” (as reported by Marla and Raquel) sustain silence. Even when children show curiosity, they often utilize functional displacement strategies, such as asking about “what happened to a friend” to avoid parental sanction. Some participants, like Lupita, attributed this absence to the child’s self-assessment of disinterest, while others, like Brenda and Lola, identified a minority trend where “ongoing dialogue” and “security” facilitate an approach. However, the prevailing macrocontingency of “family harmony” continues to favor postponement over direct interaction.

### 3.4. Reference Agents and Communicative Substitution

Family communication about sexuality does not occur in isolation; instead, it is functionally shaped by the presence of multiple social agents who participate in, regulate, or replace instructional exchanges. Mothers frequently rely on external figures, such as teachers, health professionals, and peers, to complement or substitute conversations that they perceive as difficult to initiate or sustain at home. This section examines how this reference agents operate within the family’s contingential system, highlighting patterns of instructional delegation, trust distribution, and communicative substitution that structure adolescents’ learning about sexuality beyond the household.

Family communication is functionally shaped by multiple social agents. Mothers identified a triad of influence: the mother as a moral regulator, the school as a technical agent, and peers as trust agents. There is a unanimous agreement (99%) regarding the school’s complementary role.

Participants like Julia and Maria emphasized that school is a “legitimate space” because “they explain it better there” and “young people are more inhibited with their parents.” This pattern of functional delegation acknowledges the school as a professionalized environment that can “dispel myths” (Mirna) and “make it easier to talk about taboo topics” (Paola). Ultimately, this creates a macrocontingency of educational cooperation where the school facilitates openness that the home, constrained by gender asymmetries and traditional roles, cannot consistently provide.

### 3.5. Situations That Facilitate Dialogue

Conversations about sexuality at home do not emerge spontaneously; instead, they are typically activated by contextual events that modify the conditions under which verbal behavior becomes more likely. The interviews revealed that certain everyday situations operate as antecedent stimuli that lower communicative resistance and provide opportunities, sometimes unplanned, for initiating dialogue. This section examines the environmental and social circumstances that most frequently facilitate these exchanges, highlighting how media exposure, family dynamics, and the involvement of external agents shape the functional conditions for discussing sexuality.

Conversations rarely emerge spontaneously; they are typically triggered by contextual events. Media exposure (36%), such as TV series, and family gatherings (20%) serve as the primary antecedent stimuli that lower communicative resistance. According to the participants, these moments “reduce moral tension” and allow for verbal emission.

This reliance on external triggers demonstrates that the home does not inherently produce conditions for dialogue. Instead, mothers like Carla and Mara reinforce the idea that “a professional explains it better” or that the school environment is “more trustworthy because they [the children] are more expressive there.” This describes a functional transfer of educational control where the home retains a moral validation function while the technical instruction is externalized.

### 3.6. Value Frameworks and Topic Hierarchy

Family communication about sexuality is shaped not only by immediate interactions but also by broader moral, cultural, and institutional frameworks that regulate what can be said, by whom, and under what conditions. These value systems function as macro-level contingencies that legitimize some forms of discourse while restricting others. The following section examines how mothers evaluate the role of the home and the school in sexuality education, and how their moral and normative beliefs influence the boundaries of acceptable dialogue.

While 100% of participants agree that sexual education is a shared responsibility, a clear value hierarchy exists. Topics legitimized by public health—such as contraception and STI prevention—receive positive reinforcement. In contrast, intimate topics like pleasure or sexual diversity often evoke “embarrassment” or “not knowing how” to explain them.

Lola and Mara expressed that “talking about sex is very difficult” and that they “can’t find the right words” for biological processes like erections or reproduction. This reflects a deficit in instructional repertoires rather than moral opposition. Interestingly, an emerging discursive transition toward inclusivity was observed; one mother highlighted the importance of teaching about “diverse families” to “prevent discrimination,” acknowledging that children of lesbian couples in the community deserve respect.

### 3.7. Difficult Topics and Value Hierarchies

There is a functional consensus that twelve (secondary school) is the “proper age” to begin sexual education, largely because “the body starts changing” (biological maturity) or “curiosity begins.” While a small subgroup (Lola, Marla) advocated for starting in preschool to foster “self-care and trust,” most link the onset of dialogue to the social recognition of puberty.

Regarding the eight axes of CSE, agreement levels remained high (>70%), except for the “Gender” and “Values” axes (52–55%). In these areas, religious or moral propensities sometimes functioned as contingencies of social punishment. As Tania noted, “my Christian faith does not allow” certain topics, or “there are only two genders.” However, these cases were the exception; the broader trend shows households transitioning toward a rights-based perspective, prioritizing “self-acceptance” and “prevention.”

### 3.8. Contingential Analysis of the Family Context

The family system functions as an interbehavioral field where verbal behavior is controlled by social contingencies of gender and morality. The following regularities were identified:

Microcontingency: Attempts to discuss sexuality often occur under high social exposure, increasing adolescent avoidance to preserve harmony (negative reinforcement).

Mediating History: Mothers’ own histories of censorship act as dispositional variables supporting current inhibition.

Macrocontingency: Norms of “respect” and “protection” promote the delegation of discourse to schools.

Ultimately, the family communication system shows that sexuality is addressed through reactive mediation. Dialogue is not maintained by stable educational rules but by situational contingencies. Schools and media serve as substitutive contexts, while the home fulfills a moral validation role. This confirms a functional discrepancy between “saying” (recognizing the importance of education) and “doing” (engaging in sustained instructional dialogue), as the family system prioritizes the preservation of the affective bond over technical sexual instruction.

### 3.9. Interpretative Synthesis of Functional Analysis

The analyzed family system functions as an interbehavioral field of regulation for linguistic interactions on sexuality, where behaviors of speaking or being still silent are controlled by social contingencies of gender, morality, and less frequent religion.

Mothers simultaneously assume the roles of educators and guardians of the normative order, producing an instructionally restrictive form of communication.

The primary function of dialogue about sexuality is not informational but prescriptive: to prevent behaviors perceived as risky to health, integrity, or moral image.

Verbal behavior about sexuality at home runs under a fragile balance between rules of obligation (Maria said: “one must talk about it”) and rules of avoidance (Paola said: “one should not go too deep”).

This control explains why, despite explicit recognition of sexuality education’s importance, communicative practices are still limited, fragmented, and dependent on external social reinforcement (school, media, peers), a functional discrepancy between saying and doing.

The family communication system shows adolescent mediation, where youths function as discriminative stimuli regulating sexual dialogue.

Observed functional conditions show that:

Stable educational interactions do not exist; exchanges are sporadic and conditioned by adolescents’ disposition.

No consistent differences were identified between mothers of sons versus daughters regarding the frequency of dialogue or avoidance patterns. However, a slight trend showed that conversations about pregnancy prevention were more often mentioned by mothers of daughters, whereas online information-seeking was more commonly attributed to sons.

Dialogue tends to be interrupted or avoided at the first sign of conflict or discomfort, preventing the formation of instructional verbal rules about sexuality.

Schools and media serve as primary sources of substitute learning, reinforcing the notion that the home fulfills a moral validation rather than an educational function.

Taken together, the findings describe a non-instructional interbehavioral communication system, in which sexuality is addressed under social and affective stimulus control, but without contingencies that combine functional and lasting learning.

## 4. Discussion

The aim of this study was to examine family dialogues about sexuality as contingentially regulated interbehavioral processes, identifying how caregiving roles and gender norms shape mother–adolescent communication. The interbehavioral analysis of maternal verbalizations found functional regularities describing how family practices around sexuality are organized under normative and social control systems, yet with understandable signs of cultural change and educational openness [25].

Mothers run as agents of moral and affective regulation, steering sexual dialogue toward prevention, care, and responsibility. Although avoidance or topic-shifting still appears for issues considered intimate (e.g., pleasure, reproduction), new functional dispositions toward dialogue also appear, grounded in recognizing the risks associated with silence and misinformation [26]. All participants explicitly reported wanting schools to provide sexuality education to prevent violence and adolescent pregnancy [27], as captured in remarks such as Vanesa said: “so they learn to take care of themselves and don’t go through what I did” or Jesús said: “because young people should be informed so they are not misled.” These verbalizations form emergent educational orientations that expand the functional field of family discourse.

So, the dominant macrocontingency is no longer anchored solely in moral or religious censorship but is being reorganized around protection, respect, and well-being. While household rules still reinforce prudence and thematic control, mothers acknowledge the need for comprehensive sexuality education, functionally shifting the home’s traditional role from control to collaborative education. Community leaders and moral authorities exert normative influence over what is considered acceptable discourse. As observed in the preliminary group sessions, collective disapproval operated as a social reinforcer suppressing open dialogue, indicating that moral conservatism modulates family communication about sexuality in Latin American contexts. The functional patterns identified align with the comprehensive sexuality education framework in that biological and preventive components are accepted, whereas axes related to gender, rights, and diversity evoke greater inhibition. This discrepancy highlights the importance of aligning family-based contingencies with comprehensive sexuality education principles to ensure consistent, rights-based sexual education.

The study also shows acceptance of sexual diversity as part of the educational repertoire. Discourses reflect openness to teaching about LGBTIQ+ topics and diverse families [17]; one heterosexual mother stressed the importance of school-based teaching on different family types of Maria said: “so children are not discriminated against.” Such verbalizations stand for emergent inclusive macrocontingencies, whereby social control is oriented toward preventing punishment and promoting empathy rather than repressing discourse [28].

At the microcontingency level, family interactions are characterized by moderate avoidance and functional delegation of discourse to educational contexts perceived as competent. Mothers often say that “School explains it better,” acknowledging the instructional authority of the educational system. This functional transfer of educational control reflects socially mediated learning, wherein the school acts as a substitute reinforcer; however, the family keeps a regulatory role via rules of care, respect, and prevention.

Consistent with Carpio, Irigoyen, and Varela [29], family verbal interaction is supported more by its social efficacy than by its educational effectiveness. Immediate consequences, supporting harmony and avoiding conflict, continue to modulate dialogue frequency, yet they no longer preclude a positive evaluation of sexual learning. Consequently, communicative practices adjust to more flexible coexistence rules in which information is accepted as far as it serves care and well-being functions.

Theoretically, the study corroborates the relevance of contingential analysis [29] as an explanatory system for complex educational and social phenomena. The identification of categories such as compensatory communicative avoidance, functional delegation of discourse, and adolescent mediation of dialogue shows that family interaction units are self-regulated communication fields sustained by coexistence rules and social reinforcers. The emergence of inclusive macrocontingencies (acceptance of diversity and endorsement of school-based comprehensive sexuality education) provides evidence of functional change in the moral control system and family functioning, an empirically relevant contribution for psychology and the health sciences.

Findings suggest that promoting comprehensive sexuality education should focus on modifying reinforcement conditions for family dialogue rather than instructing beliefs or values [30]. In line with Ibáñez-Bernal [31], who conceptualizes education as the functional reorganization of practices through externally defined criteria (pp. 99–101), and with Arroyo [32], effective interventions can be understood as reorganizing the interaction field, generating contingencies that foster verbalizations of openness, informed curiosity, and educational cooperation. Within this system, the family notions of gender and care are examined not as internal or motivational variables, but as normative contexts of cultural and social control. Their function is to establish criteria for moral regulation and forms of discourse that are reinforced or punished by the surrounding functional environment. Some of the observed conditions, such as community leadership, group consensus, and the management of social reputation, extend beyond the exclusive domain of Psychology, underscoring the need for an interdisciplinary approach to these phenomena [33]. From this perspective, interbehavioral psychology is conceived as a metasystem of analysis, capable of describing the functions and contingent relations among social, educational, legal, and communicative systems, and of integrating the contributions of other disciplines without dissolving into them. In doing so, the study delineates a level of analysis where sociocultural contingencies and interbehavioral processes converge to explain the functional regulation and qualitative dimensions of family dialogue about sexuality.

The literature has shown that the quality of interaction between caregivers and adolescents is a central dispositional factor in family functioning. Parent–adolescent dyads classified as conflictive display patterns of avoidance, opposition, and coercive cycles that undermine family negotiation, whereas non-conflictive dyads maintain more regulated exchanges with shorter durations of negative behaviors [34]. Their findings confirm that observable communicative practices—such as the delivery of instructions, behavioral reciprocity, or adolescent avoidance—allow for the identification of conflict levels and, consequently, for understanding the conditions that facilitate or hinder dialogue. This evidence supports the relevance of directly analyzing interactions between mothers and their children, given that adults responsible for daily caregiving typically constitute the primary source of verbal and emotional regulation within the household, regardless of the presence or absence of a paternal figure.

Building on this perspective, recent studies highlight the importance of adopting a contextual and functional analytic lens to understand the conditions under which conversations about sexuality emerge, persist, or are inhibited within families [35].

In sum, families do not oppose sexual education; they seek conditions that enable functional and ethical learning, avoiding the repetition of violence, discrimination, or silence experienced by prior generations. Comprehensive sexuality education thus emerges as a shared responsibility system in which home and school complement one another in regulating sexual discourse.

This study presents several limitations that should be considered when interpreting its findings. First, the participation of fathers was extremely low, which restricted the possibility of conducting gender-based comparisons in parental communication patterns. Second, the data were obtained through self-reported verbalizations, which may be influenced by social desirability biases. Third, the study was conducted in a single vulnerable community, which may limit the transferability of the results to other sociocultural contexts. Finally, the cross-sectional design does not allow for examining changes in communicative practices over time or across developmental stages.

## 5. Conclusions

This study shows that family communication about sexuality operates as a non-instructional interbehavioral system in which maternal verbal behavior is regulated primarily by social contingencies of gender, harmony, and moral control. The main functional finding is that dialogues emerge reactively, often triggered by media exposure, and are maintained by avoidance patterns reinforced through the preservation of family cohesion. Adolescents frequently mediate these interactions, functioning as discriminative stimuli that determine when and how dialogue can occur. These conclusions are consistent with the functional patterns observed across interviews, particularly the predominance of avoidance, adolescent mediation, and delegation to schools.

At the macro level, households exhibit a gradual transition toward inclusive value frameworks: mothers increasingly endorse school-based sexuality education and express a preventive orientation grounded in care and protection. However, caregiving norms and gendered expectations continue to restrict sustained educational dialogue.

These findings extend interbehavioral theory by demonstrating how cultural macrocontingencies and everyday microcontingencies jointly shape sexual communication in families. Practically, programs in sexuality education and public health should focus on reorganizing reinforcement contingencies, through guided materials, intergenerational dialogue exercises, and verbal-modeling strategies, to strengthen non-punitive, educational discourse at home.

Future research should incorporate additional phases of contingential analysis solution design, intervention testing, and evaluation to develop context-sensitive strategies that modify social control conditions and promote stable instructional interactions between caregivers and adolescents.

## Figures and Tables

**Table 1:** Structure of the Interview Guide.

Dimension	Category of Analysis	Questions
System of Micro-Contingential Relations System of Macro-Contingential Relations Dimension System of Micro-Contingential Relations	Demographic Data User’s Behavioral Morphologies in the Interaction	Neighborhood, age, sex, relationship with the student. Who lives at home?
		Have you ever talked about sexuality with your son/daughter on your own initiative? What do you do when a situation arises that leads to a conversation about sexuality?
	Behavioral Morphologies of Others in the Interaction Morphology of Other People	Has your son/daughter ever asked you questions about sexuality? What does your child do when a conversation about sexuality arises?
		Who else talks with your sons/daughters about sexuality? What do those people do when they talk about sexuality with your sons/daughters?
	Situations Value Framework	In what situations have you been able to talk about sexuality with your son/daughter?
		In your home, do you agree with teaching your sons/daughters topics related to sexuality? Do you agree that schools should provide sexual education to your sons/daughters? In what situations and with whom should sexuality be taught? Which topic related to sexuality is the most difficult to teach your sons/daughters? Which three topics do you consider most important regarding your son’s/daughter’s sexual education? To what extent do you agree or disagree with teaching the following topics at school to your sons/daughters? If you disagree or totally disagree with any topic, could you explain why? At what age do you consider it appropriate for students to begin receiving sexual education?
	Category of Analysis Demographic Data	Neighborhood, age, sex, relationship with the student. Who lives at home?
	User’s Behavioral Morphologies in the Interaction	Have you ever talked about sexuality with your son/daughter on your own initiative? What do you do when a situation arises that leads to a conversation about sexuality?
System of Macro-Contingential Relations Dimension System of Micro-Contingential Relations	Behavioral Morphologies of Others in the Interaction Morphology of Other People Situations Value Framework Category of Analysis Demographic Data User’s Behavioral Morphologies in the Interaction Behavioral Morphologies of Others in the Interaction	Has your son/daughter ever asked you questions about sexuality? What does your child do when a conversation about sexuality arises?
		Who else talks with your sons/daughters about sexuality? What do those people do when they talk about sexuality with your sons/daughters?
		In what situations have you been able to talk about sexuality with your son/daughter?
		In your home, do you agree with teaching your sons/daughters topics related to sexuality? Do you agree that schools should provide sexual education to your sons/daughters? In what situations and with whom should sexuality be taught? Which topic related to sexuality is the most difficult to teach your sons/daughters? Which three topics do you consider most important regarding your son’s/daughter’s sexual education? To what extent do you agree or disagree with teaching the following topics at school to your sons/daughters? If you disagree or totally disagree with any topic, could you explain why? At what age do you consider it appropriate for students to begin receiving sexual education?
		Neighborhood, age, sex, relationship with the student. Who lives at home?
		Have you ever talked about sexuality with your son/daughter on your own initiative? What do you do when a situation arises that leads to a conversation about sexuality?
		Has your son/daughter ever asked you questions about sexuality? What does your child do when a conversation about sexuality arises?

## Data Availability

The data presented in this study are available on request from the corresponding author due to ethical and confidentiality considerations.
